# Effect of Sow Body Weight at First Service on Body Status and Performance during First Parity and Lifetime

**DOI:** 10.3390/ani12233399

**Published:** 2022-12-02

**Authors:** María José Carrión-López, Juan Orengo, Josefa Madrid, Antonio Vargas, Silvia Martínez-Miró

**Affiliations:** 1 Department of Animal Production, Faculty of Veterinary Science, Regional Campus of International Excellence “Mare Nostrum”, University of Murcia, 30100 Murcia, Spain; 2 Genera PM Office, Cabo de Palos, 30370 Cartagena, Spain

**Keywords:** gilts, body status, nonproductive days, lifespan, longevity

## Abstract

**Simple Summary:**

Improving the management of the conventional pig production system is crucial to increase herd productivity. Nowadays, the reproductive efficiency of gilts and sows during their life is not as efficient as expected. Gilts’ development is related to their production efficiency. Thus, any improvement in management strategies should aim to optimize gilt development before the first insemination. Knowledge concerning the physiology of gilts, such as adequate body condition development under intensive systems, is necessary for developing proper management practices identifying gilts with the highest potential for lifetime performance. Our study showed differences in the reproductive performance of gilts grouped retrospectively according to body weight at first service. Therefore, body weight at first service could be used as a practical tool on commercial farms to track and optimize productive efficiency.

**Abstract:**

In this retrospective study, we aimed to assess the effect of body weight (BW) at first service on body status development and sow performance during first parity and lifetime efficiency. A total of 360 DanBred gilts were used, which we categorized into three groups according to their BW: (1) Small sows (<135 kg BW; n = 108); (2) Medium sows (135–150 kg BW; n = 155); (3) Large sows (>150 kg BW; n = 63). We studied the gilts from first insemination to first weaning, and then monitored until culling. Sow body status, litter size and weight, farrowing rate, weaning-to-conception interval, lifetime performance, and hormones linked to metabolism were recorded. Sows in the Small group had the lowest body weight, backfat thickness, and loin depth during gestation. Moreover, they had the smallest number of total piglets born and longest weaning-to-conception interval at first parity. The Small sows also had, on average, one less piglet born during their productive life, and they tended to have a higher culling rate over three parities. For immunoglobulins, insulin, cortisol, and IGF-I levels, no differences were found. In conclusion, achieving optimal body weight at first service is essential for maximizing the sow’s lifetime performance.

## 1. Introduction

In conventional swine production systems, improving the sow lifetime performance and longevity in the herd is an evident way to maximize productivity [1], profitability [2,3], and animal welfare [2]. Nowadays, approximately 50% of sows in breeding herds are replaced before they can reach the target parity number to be profitable [4,5,6]. Therefore, the reproductive efficiency of sows is not as efficient as expected. Accordingly, the number of gilt replacements required increases, which is associated with decreased productivity [7] and higher possibility of disrupting herd health [8]; in the latter case, either by introducing gilts with lower immunity or by increasing the chances of introducing new disease-causing microorganisms.

Gilts represent the largest category (18–20%) of females in a breeding herd [9]. Many factors, such as breed, body condition, parity, semen quality, nutrition management, and environment, can influence reproductive success [10]. Particularly, age and sow body condition (body weight, backfat thickness, and loin muscle depth) are critical for optimizing the number of born-alive piglets, subsequent reproductive success, and, consequently, for reducing early culling rates of prolific sows. However, under field conditions, the range of age and body weight of gilts at first service may be too wide. Along this line, several researchers have considered the effect of such variables. Malanda et al. [11] showed that gilts mated for the first time at 233–253 days of age were more productive either in total born piglets during their lifetime or in lower risk of being culled due to reproductive failure compared with those aged less than 233 days. Iida et al. [12] also observed that sows that were first mated at 278 days had fewer piglets born alive than those mated at an earlier age (229 days). Amaral Filha et al. [13] reported that sows with a higher growth rate from birth to first breeding (771–870 g/day), with an average body weight of 173 kg and backfat thickness higher than 17 mm, showed a higher percentage of stillborn piglets, and more piglets weighing less than 1.2 kg, as well as a higher coefficient of variation for birth weight than sows with growth rate below 701 g/day. 

In a retrospective study, Hoving et al. [14] found that relatively light and young gilts at first insemination (124 ± 0.5 kg and 230 ± 0.6 days) had lower reproductive performance in their second parity (lower pregnancy rate and litter size). Other earlier studies also established a relationship between body status and sow performance: Williams et al. [15] reported that gilts weighing less than 135 kg had fewer total pigs born over three parities than those gilts weighing more than 135 kg. However, gilts that were heavier at first breeding (150–170 kg) had a lower farrowing rate in parity two, and they were at higher risk of culling and locomotion problems over three parities [16]. On the other hand, the lack of body reserves and inappropriate body weight in feed restricted sows led to young sows being more sensitive to detrimental effects from a negative energy balance on reproduction, affecting oocyte development [17]. This could negatively affect reproductive outcomes when weight losses exceed 10–12% of initial body weight during lactation [18], being more evident in primiparous sows because they are still growing. Ensuring the good body condition status of young sows could optimize production in second parity, which were constantly reliable breeders in their subsequent litters. However, the optimal conditions for the first insemination may be related to the sow’s genetic potential.

Assessment of sow performance at first parity can predict if sows will have high healthy-productive lifetime performance [12,19]. Lifetime performance also depends on the immunological and metabolic status of the sow [20]. A further understanding of the relationship between them and their effect on the organism may lead to fewer nonproductive days, more piglets weaned per sow per year, and a lower culling rate. This might address the gap between genetic potential and lifetime productivity of sows [1]. Moreover, poor productivity and/or management was linked to inadequate age and body weight at first breeding [9]. 

Commercial farms aim to establish an optimal number of services per week, as this is one of the indicators that ensures regularity in weaned piglet production. Achieving the target number of services per week depends on the regularity of gilts entering the farm. However, gilt input is not always regular, quarantine facilities are often undersized, and pig producers are forced to inseminate sows outside the breeder’s ideal specifications. Defining specific targets for sow body weight at first service would be a suitable and practical on-farm tool to optimize sow performance. In addition, information is limited on the effects of body weight at first service for current breeding lines on reproductive performance and longevity, and how they would affect the immunological and metabolic status of these hyperprolific sows in commercial conditions. Therefore, in this retrospective study, we aimed to quantify the effect of sow body weight at first service on body status development, sow performance during first parity, and lifetime efficiency in DanBred sows. 

## 2. Materials and Methods

The experimental procedures and animal handling were approved by the Ethical Committee for Animal Experimentation of the University of Murcia and the Administrative Authorities (A-13170805), we conducted the study in accordance with the European guidelines for the protection of animals used for scientific purposes [21].

### 2.1. Animals 

A total of 360 DanBred (Landrace x Large-White) gilts from a commercial farm located in Murcia, Spain, were used in this study (from April 2018 to February 2022). At the beginning of the study, gilts were housed in the gilt development unit; after insemination, they were moved to the gestation unit. 

### 2.2. Puberty Stimulation

Skilled farm workers checked sows to detect first estrus at 24 h intervals in the presence of an epididiectomized boar. Every day in the morning, pens of gilts received 20 min of direct exposure to a boar (before first estrus was detected). On the first day of standing reflex in response to the back-pressure test, sows were marked and recorded. Then, active progestin, Altrenogest (Regumate^®^, Merck, White House Station, NJ, USA) was orally administered to gilts every Wednesday and for 18 consecutive days to synchronize estrus and ensure the target number of services per week. On the other hand, gilts not responding to puberty stimulation for approximately 2 months were sent to slaughter and were not included in further analysis.

### 2.3. Breeding and Management

In the gilt development unit, females were housed in groups of 16 each on partially slatted floors and with a space allowance of at least 1.30 m^2^. All gilts were allowed ad libitum access to water and feed. When gilts showed pubertal estrus, they were removed from the original pen group and relocated for acclimatization in the gestation crates (2.40 m × 0.60 m) with partially slatted floors. At approximately 25 days from the first detection of estrus, gilts were moved to gestation facilities and allowed fenceline contact with mature boars for the detection of the second estrus. Once on the day of showing strong estrus, and every 24 h during the standing heat period, gilts were inseminated by artificial cervical insemination with Duroc semen at a dose of 3.0 × 10^9^ morphologically normal sperm. All gilts were also allowed contact with a mature boar during insemination. Gilts were kept until day 28 of gestation, when they were moved to indoor pens (7.0 m × 5.5 m) and kept loose in groups of 16 each. Females were often supplied with straw; in addition, in loose-housed, a wooden block was provided to each pen. 

On day 110 of gestation, gilts were moved into farrowing crates (0.60–0.80 m × 2.20–2.40 m according to the sow weight), with a rubber-coated expanded-metal floor. Alongside the farrowing crate, within the pen, was an enclosed and heated creep area for the piglets. The piglets were managed in accordance with the general routines on farm: all piglets were tail-docked, and received an iron injection (Uniferon^®^, Pharmacosmos, Holbaek, Denmark) and an injection against coccidiosis (Baycox^®^, Bayer Animal health division, Leverkusen, Germany) at 72 h postpartum. On this farm, tail docking was performed due to the problems that occurred during the nursery period; despite changes implemented in handling and facilities (such as decreasing animal density, ensuring adequate environmental conditions according to piglets’ age and weight, and incorporating enrichment materials). Then, piglets were cross-fostered to standardize litters to 14 piglets. At weaning (25 ± 4 days of lactation), they returned to the individual gestation crates. All measurements and management practices were performed according to European [22] and local law [23]. 

### 2.4. Feed Management

Gilts in the development unit were fed a gilt-developer diet (3182 Kcal DE/kg, 134.6 g CP/kg, and 8.4 g lysine/kg), offered ad libitum from approximately 21 weeks of age until the first estrus. During the entire estrus synchronization treatment (18 days), they were fed at a rate of 2.2 kg/day until they were inseminated. Then, a commercial gestation diet was supplied (2982 Kcal DE/kg, 125.1 g CP/kg, and 6.6 g lysine/kg), which was increased to 3.0 kg/day until day 28 of gestation according to body condition score. From day 29 to 110, the feeding level was again decreased to 2.2 kg/day. From day 110, the animals were fed the same amount of a conventional commercial lactation diet (3174 Kcal DE/kg, 162.4 g CP/kg, and 10.4 g lysine/kg). After farrowing, sows were fed three times a day: at 07:00, 12:00, and 18:00 h. The initial amount of feed was adjusted for each lactating sow by gradually increasing the daily amount supplied by 0.5 kg/day until a maximum of 10 kg/day. From weaning and during next productive cycles, all sows were offered a gestation diet of approximately 3.0 kg/day. The diets were based on wheat, barley, maize, and soya, and were offered in pellet form. Piglets were offered a commercial creep feed (3420 Kcal DE/kg, 177.0 g CP/kg, and 14.0 g lysine/kg) from 7 days of age until weaning (25 ± 4 days of age). Feed intake per sow was not recorded. 

### 2.5. Experimental Design: Body Weight and Age Groups at First Service

When the estrus synchronization treatment was finished, the body weight (BW) and age of gilts were recorded. Those gilts that did not show estrus within 8 days after synchronization treatment were omitted from the analysis (n = 34). Then, gilts exhibiting estrus were retrospectively categorized into three groups of body weight: 1) Small sows (<135 kg BW; n = 108); 2) Medium sows (135–150 kg BW; n = 155); 3) Large sows (>150 kg BW; n = 63). The three categories of BW at first service were established according to the limits close to the values observed in the distribution of the 30th and 80th percentiles. In addition, we also created three age groups based on the same percentiles (30th and 80th) according to gilt age at first service: 210 days or earlier (n = 96), from 217 to 231 days (n = 166), and 238 days or older (n = 64). We studied the gilts from first insemination to first weaning, and then monitored them until their culling.

### 2.6. Measurements

#### 2.6.1. Sow Development

The records for each sow comprised sow identities, birth date, BW, body condition score (BCS), backfat thickness (BFT), and loin muscle depth (LD). Sow BW was recorded at insemination considered as day 0 (d0), day 110 (d110) of pregnancy, and at weaning (dwe). Other body data, such as BCS, BFT, and LD were recorded at day 28 (d28), 60 (d60), and 90 (d90) of pregnancy. The BCS was always evaluated by the same person on an ordinal scale from 1 (very thin) to 5 (very fat) according to Bonde et al. [24]. The BFT and LD were measured by ultrasound scan equipment with a linear probe (SF1, Wireless Backfat and Loin Depth Scanner, Sonivet, Beijing, China) at the P2 position (last rib, 65 mm from the center line of the back). Within each control day, the ultrasound measurements were repeated four times (twice on each side), and their average was used for further calculations.

#### 2.6.2. Sow Performance

For the first litter, the number of total piglets born (NTB), number of piglets born alive (NBA), number of piglets stillborn (NSB), number of piglets mummified (NMM), piglets’ birth weight within 24 h (BW24), piglets’ mortality rate at 72 h post-partum (MR72), and number of weaned piglets (NWP) were recorded. The MR72 was calculated as the percentage of dead relative to alive born during first 72 h post-partum. Subsequently, the weaning-to-conception interval (WCI) after first weaning was also recorded. 

From the second cycle onward, records from each sow and its litters were extracted from the farm management software (developed by the company in-house and made in Windows Form with C#). Data included the average of total piglets born over their lifetime, percentage of sows completing three parities, total piglets weaned over three parities, percentage of sows producing 40 piglets weaned over three parities, number of days from birth to culling, average parity number at culling, number of births per insemination, and average WCI. Sows were culled according to standard commercial practices. Age and reason for culling were also recorded. The main causes of culling were reproductive failure (failure to exhibit a pubertal estrus, failure to conceive, or abortion), poor performance (repeated twice in a row, dystocia, lack of milk supplied by the sow, clinical mastitis, or illness), locomotion problems (lameness), or death.

### 2.7. Blood Samples and Hormone Analysis

For blood collection, 80 sows were randomly sampled from the total number of pregnant sows in the study (Small (n = 32), Medium (n = 32) and Large (n = 16) sow groups). Blood samples were taken from the cranial vena cava in sterile tubes without additives (Vacuette^®^, Greiner Bio-One, Kremsmünster, Austria) to determine metabolic hormone status at day 110 of pregnancy. For this purpose, sows’ movements were restrained with a snare by skilled personnel to ensure blood extraction in a short period of time. Blood samples were centrifuged at 1600× *g* for 10 min; then, serum was stored at –80 °C until further analysis of the levels of immunoglobulins (IgM, IgG, and IgA), insulin, cortisol, and insulin-like growth factor-1 (IGF-1). 

We submitted the serum samples to the Interdisciplinary Laboratory of Clinical Analysis of the University of Murcia (Interlab-UMU, Spain). All immunoglobulins were analyzed with commercial kits for pigs (reference E101-104; E101-117; and E101-102, respectively) (Bethyl Laboratories, Inc., Montgomery, TX, USA). We analyzed cortisol and IGF-1 levels with an automated chemiluminescent immunoassay (Immulite System, Siemens Health Diagnostics, Deerfield, IL, USA). Plasma insulin was determined by Enzyme-Linked ImmunoSorbent Assay (ELISA) Kit (Mercodia, Porcine Insulin ELISA ref. 10-1200-01, Winston Salem, NC, USA).

### 2.8. Statistical Analysis

All analyses were performed using Statistics software SPSS version 28.0 for Windows (SPSS Inc., Chicago, IL, USA). First, descriptive statistics were examined for the groups of body weight (BW) and age at first service of gilts, including the correlation between the two variables (Pearson’s coefficient). Preliminary analysis of the data considered both factors, weight and age of gilts at first service (with three groups or levels within each, as noted above), as well as the interaction between them. However, age and the first interaction effect were mostly not significant and, therefore, they were omitted from further analysis. The data analysis concerned only gilts after receiving Altrenogest. The 34 females that did not show the second controlled estrus were not inseminated (7, 5 and 23 for Large, Medium, and Small, respectively), and were only taken into account when calculating the percentage of sows bred within 8 days after synchronization treatment.

Body measurements of the gilts on different days throughout the study were analyzed using a mixed model. The model included the factors of gilt weight (Small, Medium, and Large gilts) as a fixed effect, and sow as the random effect (repeated measures). Additionally, Pearson’s coefficient was determined to assess the relationships between BCS and BFT, and BCS and LD.

Litter size (NTB, NBA, NSB, and NMM) and piglet weight (BW24) at first birth were analyzed with an ANOVA model, in which we included gilt weight as the main factor, and the NTB as a covariate. Gilts that returned to estrus after cervical artificial insemination were not used for analyzing data from the first litter (n = 25). The number of weaned piglets (NWP) at first birth and variables linked to lifetime performance (average of total piglets born, total piglets weaned over three parities, number of days from birth to culling, and average parity number at culling) were also analyzed using the same model but without covariate.

Other variables, such as the proportion of sows bred within 8 days after estrus synchronization treatment, farrowing rate, culling rate at first parity, causes of culling, percentage of sows completing three parities, and percentage of sows producing 40 piglets weaned over three parities were coded as binary variables for each sow, which we then analyzed by logistic regression and estimated within the weight group at first service. Therefore, a binomial distribution (binary response) with a logit model was fitted to evaluate them, where gilt weight was used as independent variable. The number of births per insemination was also analyzed by logistic regression, as the number of events occurring in a set of trials. However, the weaning-to-conception interval and variables linked to hormonal status at 110 days of gestation (immunoglobulins (IgM, IgG, and IgA), insulin, cortisol, and IGF-1 levels), which showed non-normal distribution, were analyzed with nonparametric tests (median and Kruskal–Wallis tests). 

The gilt (or its litter) was considered the experimental unit according to the variable analyzed. If normally distributed, results are presented as means, whereas those not normally distributed are presented as medians. A pairwise comparison of means was performed by using the least significance difference (LSD) test. The significant value was set at *p* ≤ 0.05, while 0.05 < *p* ≤ 0.10 was considered as a tendency. 

## 3. Results

Sow body weight at first service was moderately correlated with age (r = 0.539; *p* < 0.001; Table 1). However, when weight groups were taken into account, no significant effect of gilt age groups was found for most of the tested variables. Therefore, the results shown concern only the effect of the BW of gilts.

### 3.1. Effect of Body Weight at First Service on Body Condition Score, Backfat Thickness, Loin Depth, and Body Weight through Gestation and Lactation of First Parity

**Figure 1:**
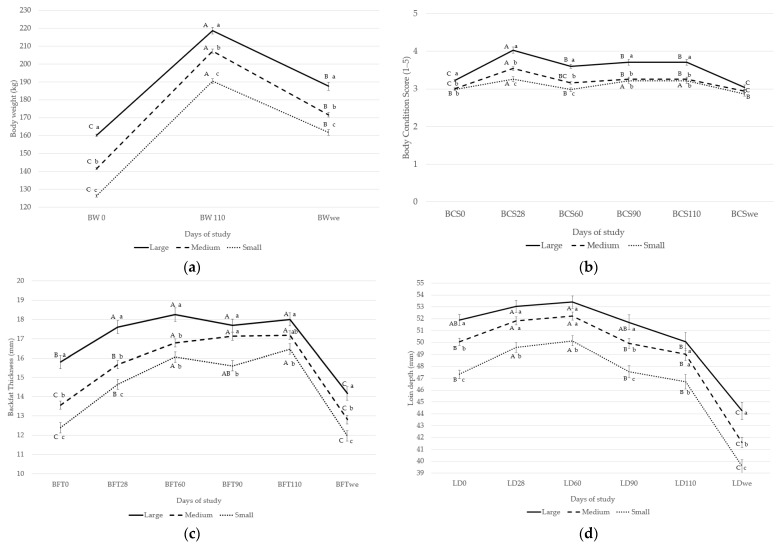
Effect of body weight of gilts at first service on evolution of (**a**) body weight (BW), (**b**) body condition score (BCS), (**c**) backfat thickness (BFT), and (**d**) loin depth (LD) during first parity and at weaning (we). The BCS was evaluated using an increasing ordinal scale from 1 (very thin) to 5 (very fat) according to Bonde et al. [24]. Lowercase letters represent differences between groups at each time point. Capital letters represent within-group differences over time. Weight group at first service: (1) Large sow (>150 kg BW; n = 63); (2) Medium sow (135–150 kg BW; n = 155); (3) Small sow (<135 kg BW; n = 108).

The BW at first service (d0) differed between groups (*p* ≤ 0.05), with means of 126.3, 141.6, and 160.1 kg for the Small, Medium, and Large groups, respectively (Figure 1a). At 110 days of gestation and at weaning, the differences between groups were maintained (*p* ≤ 0.05). When BW changes over time were evaluated for each group of sows, it was observed that the sow weight increased from day 0 to 110; however, from day 110 to weaning, it decreased.

At day 0, sows in the Small or Medium group showed a lower BCS than those in the Large group (*p* ≤ 0.05) (Figure 1b). These differences were maintained at days 90 and 110. At days 28 and 60, the BCS of the sows in the Small group were lower than in the Medium group, and the BCS of the sows in the Medium group were lower than that of the sows in the Large group (*p* ≤ 0.05). At weaning, no differences were found among the three groups (*p* > 0.05). The BCS of the three weight groups increased during the first 28 days, then decreased and remained constant until day 110 of gestation, except for in the Small group, where BCS increased again on day 90. At weaning, the BCS decreased to values similar to those of day 0 in all groups. 

The BFT at days 0 and 28 differed among the three groups, with sows in the Small group showing the lowest average, followed by sows of the Medium and Large groups (*p* ≤ 0.05; Figure 1c). These differences in favor of the Large group were maintained throughout the experiment, except at days 90 and day 110, where the BFT of the sows of the Medium group did not differ from that of the Large group. The BFT of all sows increased from day 0 to day 110, then their fat reserves reduced during lactation. 

From day 0 to weaning day, the LD values of the sows in the Small and Large groups were the lowest and highest, respectively (*p* ≤ 0.05; Figure 1d). Sows in the Medium group had intermediate LD values at days 0 and 90 and at weaning (*p* ≤ 0.05). At days 28, 60, and 110, the values were similar to those of the sows in the Large group (*p* > 0.05). On average, the LD increased until reaching a maximum at day 60; however, it decreased from this day onward. The lowest data were recorded at weaning.

In addition, low correlations were found between BCS and P2 BFT (r = 0.30, *p* < 0.001) and between BCS and LD measurements (r = 0.31; *p* < 0.001).

### 3.2. Effect of Body Weight at First Service on Reproductive Efficiency during First Parity

Table 2 shows the results for the litter performance parameters from first insemination to first weaning. Regarding litter size, NTB was 1.5 and 1.0 piglets lower in the Small group than in the Large and Medium groups, respectively (*p* ≤ 0.05). However, there were no differences between the sow size groups for NBA, NSB, and NMM (*p* > 0.05); nevertheless, we observed a tendency in which sows in the Large group had a lower NMM (*p* ≤ 0.10). The MR72 did not differ among the three groups (*p* > 0.05). Likewise, neither the BW24 nor the NWP differed between the weight groups of gilts (*p* > 0.05).

### 3.3. Effect of Body Weight at First Service on Estrus Detection within 8 Days after Synchronization Treatment, Farrowing Rate, Culling Rate, and Weaning-to-Conception Interval at First Parity

**Figure 2:**
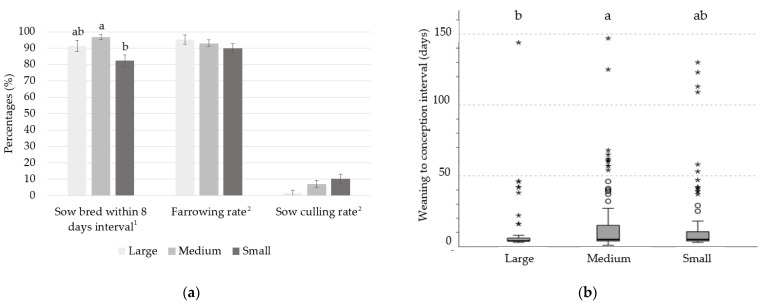
Effect of body weight of gilts at first service on (**a**) proportion of sows bred within 8 days after estrus synchronization treatment, farrowing rate, and sows’ culling rate; (**b**) weaning-to-conception interval at first parity. ^1^ Weight group at first service: (1) Large sow (>150 kg BW; n = 69); (2) Medium sow (135-150 kg BW; n = 160); (3) Small sow (<135 kg BW; n = 131). ^2^ Weight group at first service: (1) Large sow (>150 kg BW; n = 63); (2) Medium sow (135–150 kg BW; n = 155); (3) Small sow (<135 kg BW; n = 108). Lowercase letters represents statistically significant differences (*p* < 0.05). °, *: outliers are identified with different markers for “out” (small circle) and “far out” (marked with a star), the latter SPSS also calls “extreme values”.

The percentage of sows bred within 8 days after synchronization treatment was 14 points lower in the Small group than in the Medium group (82.44 vs. 96.88; *p* ≤ 0.05); whereas an intermediate proportion was found in the Large group (91.30%), which showed a tendency to be higher than in the Small group (*p* ≤ 0.10) (Figure 2a). However, from the total of sows that were inseminated, the percentage of sows that farrowed did not differ among the three groups (ranging from 89.81% to 95.24% for the Small and Large groups, respectively; *p* > 0.05) (Figure 2a). 

**Figure 3:**
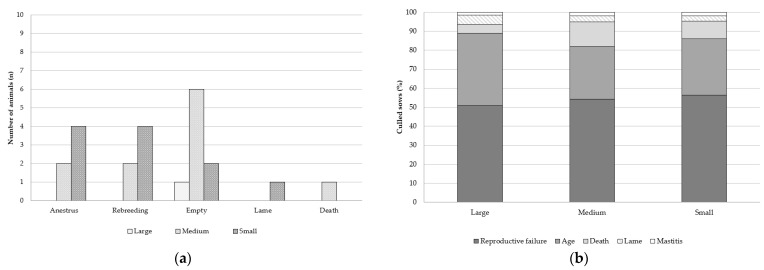
Effect of body weight of gilts at first service on (**a**) reasons of removal at first parity or (**b**) during their lifetime (expressed as absolute and relative frequencies, respectively). Weight group at first service: (1) Large sow (>150 kg BW; n = 63); (2) Medium sow (135–150 kg BW; n = 155); (3) Small sow (<135 kg BW; n = 108). No differences in reasons for culling throughout their lifetime were detected between weight groups of gilts (*p* > 0.05).

When sows were weaned, the culling rate did not differ among the three groups (*p* > 0.05; Figure 2a). The main reason for culling at first parity was reproductive failure (anestrus, rebreeding, or empty after gestation ultrasound diagnosis; Figure 3a).

Figure 2b presents the results for weaning-to-conception interval (WCI) after first weaning for sows in the Large, Medium, and Small groups. The data distribution according to the Kruskal–Wallis test differed among the groups (*p* ≤ 0.05), with sows having lower clustered data in the Small and Medium groups than in the Large group. Thus, the median days from weaning-to-conception decreased when BW at first service increased, such that WCI was 5.0, 5.0, and 4.0 days for the Small, Medium, and Large groups, respectively. 

### 3.4. Effect of Body Weight at First Service on Reproductive Efficiency and Their Lifetime Performance

Table 3 shows the effect of BW at first service of sows on the lifetime performance parameters on a commercial farm. The average of total piglets born over the lifetime increased as BW at first service increased (*p* ≤ 0.05). Across the three first cycles, we found a tendency of a lower percentage of sows completing three parities in the Small and Medium group than in the Large group (*p* ≤ 0.10). However, the average of total piglets weaned over three parities did not differ among groups (*p* > 0.05). Furthermore, the percentage of sows producing 40 piglets weaned over three parities tended to be lower in the Small or Medium group than in the Large group (*p* ≤ 0.10).

The number of days from birth to culling was lower in the Small or Medium groups than in the Large group (*p* ≤ 0.05). However, the average parity at culling did not significantly differ among the three groups; nevertheless, sows in the Small group numerically had approximately one cycle less than sows in the Large group (5.87, 6.03, and 6.73 parities for Small, Medium, and Large sows, respectively; *p* ≤ 0.10). Moreover, there was no association between the weight at first service and the reason for sow culling (*p* > 0.05; Figure 3b). The most common reasons in all groups were reproductive disorders (53.8%) followed by age, death, lameness, and mastitis (31.8%, 9.0%, 3.6%, and 1.8%, respectively).

The average percentage of births per insemination was 89.77% without differences between the weight groups of gilts (*p* > 0.05). Additionally, the distribution of the average WCI between the weight groups of gilts was not affected by the body weight at first service (*p* > 0.05), showing a global median of 5.13 days.

### 3.5. Effect of Body Weight at First Service on Serum Immunoglobulin, Leptin, Cortisol, and IGF-1 Concentrations of Gilts on Day 110 of Gestation

**Figure 4:**
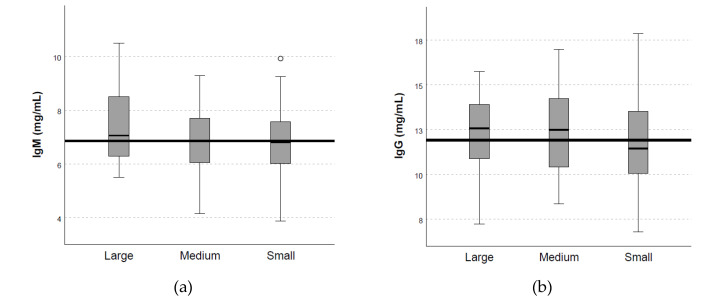

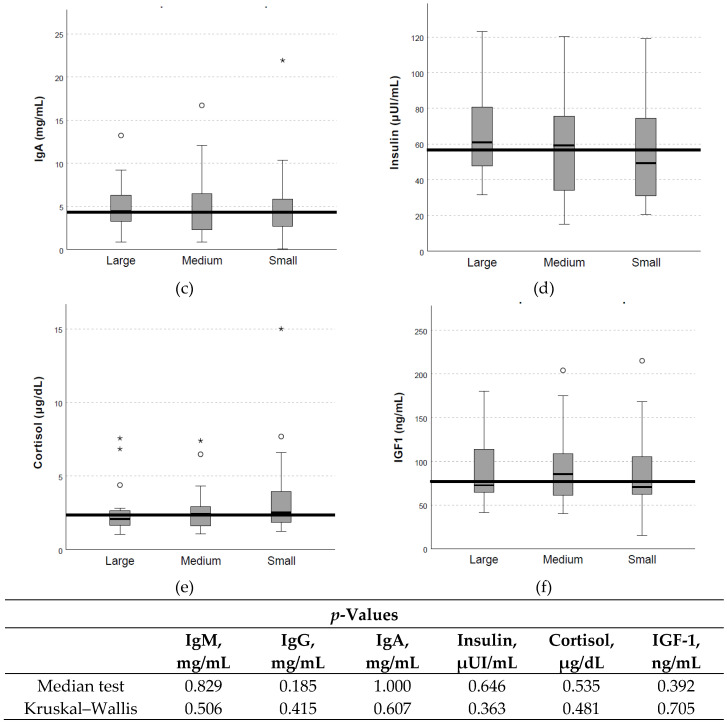
Effect of body weight at first service on serum immunoglobulin concentrations: (**a**) IgM, (**b**) IgG, (**c**) IgA, (**d**) insulin, (**e**) cortisol, and (**f**) IGF-1. Weight group at first service: (1) Large sow (>150 kg BW; n = 16); (2) Medium sow (135–150 kg BW; n = 32); (3) Small sow (<135 kg BW; n = 32). Solid horizontal line represents global median for each blood metabolite. No significant differences were detected between weight groups of gilts (*p* > 0.05). °, *: outliers are identified with different markers for “out” (small circle) and “far out” (marked with a star), the latter SPSS also calls “extreme values”.

Serum IgM, IgG, IgA, insulin, cortisol, and IGF-1 concentrations were not influenced by BW at first service (*p* > 0.05; Figure 4). The global medians were 6.86 mg/mL, 11.92 mg/mL, 4.31 mg/mL, 56,66 µIU/mL, 2.37 µg/dL, and 77.05 ng/mL for IgM, IgG, IgA, insulin, cortisol, and IGF-1 concentrations, respectively. 

## 4. Discussion

Early identification of gilts with the greatest capacity to develop a high productive potential is crucial to improving farm productivity. The BW at first breeding impacts productive performance. Thus, several pig breeding companies recommend an optimal BW at the first breeding. For example, PIC recommends that 90% or more of gilts be inseminated between 135 and 160 kg. However, Topigs proposes achieving a BW between 150 and 170 kg prior to insemination, while DanBred recommends an intermediate interval for optimum BW (140–160 kg). All these ranges are wide and may determine differences for sows with weights close to the limits of these intervals. Furthermore, from a practical point of view, to guarantee the weekly flow of weaned piglets, farmers must reach the target number of services per week (with approximately 20% of gilts). To achieve this purpose, farmers often decide to inseminate sows below the recommended body weight specifications, which may impact their productive performance.

### 4.1. Effect of Body Weight at First Service on Body Condition Score, Backfat Thickness, Loin Depth, and Body Weight through Gestation and Lactation of First Parity

The results of this study showed that the BW of sows increased during the gestation period independent of BW at first breeding. However, the BW gain of the sows in the Small and Medium groups was larger than that in the Large group (51% and 46% vs. 37%, respectively). In addition, the sows in these first two groups did not reach the optimal BW recommended by Kim et al. [25], who showed that ensuring 210 kg at day 109 of gestation leads to the lowest culling rate and highest number of piglets born alive over six parities. Sows with a lower BFT showed higher BFT gain (14.0% vs. 27.5% and 33.8% for the Large, Medium, and Small groups, respectively). As for LD, sows in all groups showed the same evolution during the gestation period. Sows deposited protein mass during the first 60 days, then mobilized it, reaching values similar to those of day 0 on day 110.

As expected, the BW of the sows in all groups decreased during lactation, when they showed similar losses. However, sows in the Large group mobilized more adipose tissue. The higher BFT losses in the Large group could be explained by a higher negative energy balance during lactation, as detailed by Maes et al. [26] and Houde et al. [27]. Along this line, Kim et al. [25] observed that during the lactation period, BFT losses linearly increased with the increase in BW at the end of gestation for parity 1. Regarding LD, sows in all groups mobilized the same quantity of protein mass. These results are consistent with the observations of Young et al. [28], who reported that the reproductive period of sows was associated with high energy requirements, especially during the last third of gestation and during lactation. Thus, the sows require high levels of nutrient intake during late gestation for both its fetuses and self-growth, leading to the mobilization of protein and fat reserves.

Sows in the Large group showed the highest BCS at the beginning of the study. However, the three groups had similar BCS at weaning day. From the perspective of using BCS to adjust feed allowances during gestation on a commercial farm, our results showed that the changes in BFT and LD without changes in BCS may have important implications. Thus, BCS appears to be a poor tool indicating the actual adipose and protein tissue mobilization during gestation and lactation periods. This is supported by the low correlation found between BCS and P2 BFT or BCS and LD measurements, as previously described Maes et al. [26]. Therefore, commercial feeding practices only related to sow body condition do not ensure that the maintenance needs of the modern sow are met.

### 4.2. Effect of Body Weight at First Service on Reproductive Efficiency during First Parity

All gilts subject to be inseminated on-farm were included in the trial. Thus, only when first estrus was detected, they were synchronized regardless of age. In any case, the age at first service of sows in the Small group was in the optimal age (close to 210 days) according to Serenius and Stalder [29]. In addition, the upper age limit suggested by Iida et al. [12] for first service of 278 days was not reached.

Our findings showed that BW at first service influenced litter size (NTB). Thus, sows in the Small group had 1.5 fewer piglets than Large gilts, in accordance with the results reported by Amaral Filha et al. [13] and Kim et al. [25]. The latter authors observed that sows that reached 210 kg on day 110 of gestation were those with higher prolificacy. However, no differences among sow weight groups were found in either piglets born alive at birth, birth weight, or mortality rate (NBA, BW24, and MR72); even though NBA was numerically higher in sows of the Large group, and the number of piglets mummified (NMM) tended to be lower. In this regard, genetically increasing the NBA decreases piglet quality, due to lower birth weight, and increases preweaning mortality [30]. Weaned piglets (NWP) were also not significantly affected by the sow BW at first service, in agreement with the results found by Lee et al. [31], who used sows of similar weight to ours.

Therefore, heavier sows had higher initial body reserves and reproductive efficiency at first parity. This could be explained by the gilts bred at a higher BW having higher reproductive maturity, which in turn could have been associated with a higher ovulation rate and a lower embryonic or fetal mortality rate.

### 4.3. Effect of Body Weight at First Service on Estrus Detection within 8 Days after Synchronization Treatment, Farrowing Rate, Sow Culling Rate, and Weaning-to-Conception Interval at First Parity 

The results of this study showed that 9% and 14% more sows in the Large and Medium groups compared with the Small group were in estrus within 8 days after synchronization treatment. These results do not agree with those reported by Thitachot et al. [32], who observed longer intervals to estrus in heavier gilts. They explained that the relationship may have been due to a larger reservoir of Altrenogest in the adipose tissue in fatter gilts. However, the heavier gilts in that study had considerably more BFT (more than 17 mm) than the gilts in our study. Nevertheless, BW at first service did not influence farrowing rate, suggesting that when gilts were synchronized and showed estrus, their reproductive system was adequately mature, so successful artificial insemination was ensured. 

As in previous studies, a high proportion of culled sows were unplanned [33]. A high rate of premature culling of sows from the herd occurs at first parity, most of them attributed to reproductive disorders [34,35], which is in line with that observed in this study (anestrus, rebreeding, or empty sows). Despite the lack of significance, the culling rate of sows at first parity was 8.6% higher in the Small group than in the Large group. Sows in the Large group also showed the shortest WCI after first weaning. In this sense, an increase in the number of nonproductive days leads to a decrease in the productive performance of sows and, therefore, a higher risk of removal [4,35,36]. Moreover, the second parity dip is associated with lower reproductive performance, lower farrowing rate, and higher returns [37], leading to an extended weaning-to-estrus interval. Hoving et al. [14] described an effect of BW on weaning-to-estrus interval: that lighter gilts (124 kg) at first insemination showed a 2-day increase in the weaning-to-estrus interval for the second cycle compared with heavier gilts (145 kg). The reason may be that if mature growth is not achieved during the first cycle, the gilt might prioritize growth over reproduction after weaning, which may result in nonpregnancy at second parity. 

### 4.4. Effect of Body Weight at First Service on Reproductive Efficiency and Their Lifetime Performance

Lighter sows at first insemination had one less piglet born than heavier sows during their lifetime. This result supports the hypothesis that NTB at first parity is an early and powerful predictor of sow prolificacy in later parities, as described by Koketsu and Iida [38], which suggests that prolificacy is another marker of lifetime performance. Thus, the results of this study showed that the performance of a sow during her first litter provides insight into the rest of her productive life. This finding may be related to animal welfare, health, and an adequate body condition status, because healthy sows are more able to handle the physiological stress and requirements of piglet production over numerous parities [8]. 

Hoge and Bates [2] determined the relationship between first parity performance and developmental factors with longevity using hazard models. They found that fatter sows at first parity tended to have a decreased risk of being culled during their productive life. These findings are consistent with our results, where about 18%, 14%, and 5% of Small, Medium, and Large sows were removed from the herd before reaching three parities. Thus, sows in the Small group tended to be culled at earlier parities and showed a lower average parity in the herd. Englom et al. [4] suggested that sows become profitable from third parity onward; therefore, a higher percentage of lighter sows were culled without producing an economic benefit and without being able to meet the reproductive goals. However, sows with excessive fat reserves (18–23 mm) [16] or excessive body weight (>170 kg) [39] at first farrowing may have an increased risk of being culled. 

The decision to remove a sow from the herd is based upon criteria such as sow parity, production level, reproductive status, and clinical health status, as well as herd structure and access to replacement gilts. Regardless of sow weight group, the current results showed that the highest proportion of sows were removed as a result of reproductive failure, followed by age, death, lameness, and mastitis, in agreement with the findings of previous studies [33,35,40].

### 4.5. Effect of Body Weight at First Service on Serum Immunoglobulin, Leptin, Cortisol, and IGF-1 Concentrations of Gilts on Day 110 of Gestation

The use of hyperprolific sow lines has led to an increase in litter size. The increase in the NBA extends farrowing duration and decreases the average BW of piglets and their viability, increasing the competition for colostrum intake and negatively affecting piglet survival [41]. The maternal immunoglobulins are transferred to piglets via colostrum [42]. Thus, a higher level of immunoglobulins in the serum at the end of gestation may enhance the transfer of passive immunity from the sow to the neonate, improving its subsequent development. Additionally, different hormones are related to sow performance and body status. Positive correlations have been described between cortisol in late gestation and litter size [43], between cortisol concentrations and average piglets BW at birth [44], and between insulin and litter size [45] in gilts. Another hormone described as playing a role in the regulation of growth and body composition was IGF-1 [46]. The increased insulin and IGF-I levels around parturition were able to mobilize enough energy from the body reserves to prevent metabolic disorders, even during a period with deficient energy supply [47]. 

Despite these positive results, immunoglobulins, insulin, cortisol, and IGF-I concentrations at day 110 of gestation were not associated with BW at the first service of gilts. To the best of our knowledge, studies on the effect of body weight at first service on immunological and nutritional parameters are limited. Thus, further longitudinal research is needed to investigate the changes in these biomarkers throughout the pregnancy, peripartum, and lactation periods.

## 5. Conclusions

Sow body weight and age at first service have been described as critical for optimizing performance at first parity, and subsequent reproductive success. Our findings showed a significant effect of body weight rather than age. Nevertheless, retrospectively grouping DanBred sows according to their BW or age at first insemination was a result of the combination of other factors (e.g., feed intake, health status, reproductive maturity, silent estrus, etc.). Gilts below the recommended optimum weight at first service were associated with lower productive performance in their first parity and lifetime performance. Thus, the introduction of gilts into the herd at an optimal body weight at first service is essential. Furthermore, body weight at first service can be also used as an early indicator of subsequent reproductive success in sows, including minimizing sow culling rate and the number of nonproductive days. In summary, body weight at first service may be used as a practical tool at the commercial level to monitor and optimize the productive efficiency of a herd. Further studies are needed to clarify whether a delay in mating smaller gilts would improve lifetime performance, or whether lower body weight is an indicator of poor overall development, including reproductive development.

## Figures and Tables

**Table 1:** Descriptive statistics of gilts according to body weight (BW) and age at first service (days from birth to first service).

	Body Weight ^1^ (kg)	Age ^1^ (d)
Weight groups (kg)		
Small (<135 kg BW; n = 108)	126.3 ± 0.7 (104.5–134.5)	214.2 ± 1.2 (196.0–252.0)
Medium (135–150 kg BW; n = 155)	141.6 ± 0.5 (135.0–149.5)	222.9 ± 1.1 (196.0–266.0)
Large (>150 kg BW; n = 63)	160.1 ± 1.1 (150.5–179.5)	235.1 ± 2.1 (203.0–294.0)
Age groups (d)		
210 d or earlier (n = 96)	132.2 ± 1.2 (108.5–159.5)	205.0 ± 0.6 (196.0–210.0)
217 to 231 d (n = 166)	140.4 ± 0.8 (104.5–167.0)	223.3 ± 0.4 (217.0–231.0)
238 d or older (n = 64)	151.0 ± 2.0 (109.0–179.5)	246.1 ± 1.3 (238.0–294.0)

^1^ Means ± SEM (range of values are shown within parentheses). BW: Body weight.

**Table 2:** Effect of body weight of gilts at first service on reproductive performance parameters from first insemination to first weaning on a commercial farm (NTB: number of total piglets born; NBA: number of piglets born alive; NSB: number of piglets stillborn; NMM: number of piglets mummified; BW24: piglet´s birth weight within 24 h; MR72: piglet mortality rate at 72 h postpartum; NWP: number of weaned piglets).

	Weight Group ^1^			SEM ^2^	*p*-Value
	Large	Medium	Small		
Sample size (n)	60	144	97		
NTB (n)	17.83a	17.35a	16.31b	0.208	0.012
NBA ^3^ (n)	16.00	15.51	15.88	0.107	0.109
NSB ^3^ (n)	1.01	1.30	1.01	0.100	0.318
NMM ^3^ (n)	0.10	0.31	0.22	0.038	0.091
BW24 ^3^ (kg)	1.21	1.20	1.18	0.010	0.652
MR72 ^4^ (%)	11.03	12.33	12.58	0.484	0.448
NWP (n)	14.81	13.51	13.91	0.289	0.201

^1^ Weight group at first service: (1) Large sow (>150 kg BW; n = 63); (2) Medium sow (135–150 kg BW; n = 155); (3) Small sow (<135 kg BW; n = 108); ^2^ SEM: standard error of the mean; ^3^ Adjusted means: the number of total born was used as a covariate for these variables in the statistical model; ^4^ Data analyzed by logistic regression. a, b Means within a row with different letters were significantly different at *p* ≤ 0.05.

**Table 3:** Effect of body weight of gilts at first service on longevity parameters over their lifetime on a commercial farm.

	Weight Group ^1^			SEM ^2^	*p*-Value
	Large	Medium	Small		
Sample size (n)	63	155	108		
Total piglets born ^3^ (n)	18.49a	17.90ab	17.42b	0.159	0.044
Sows completing three parities ^5^ (%)	95.24	85.81	82.41	2.191	0.078
Piglets weaned over three parities (n)	42.88	41.25	39.90	0.584	0.159
Sows producing 40 piglets ^4,5^ (%)	90.48	78.06	76.85	2.481	0.082
Days from birth to culling (d)	1187a	1070b	1040b	22.692	0.045
Parity at culling(n)	6.73	6.03	5.87	0.154	0.098
Number of births per insemination ^5^ (%)	91.23	88.22	89.85	0.007	0.188

^1^ Weight group at first service: (1) Large sow (>150 kg of BW; n = 63); (2) Medium sow (135–150 kg of BW; n = 155); (3) Small sow (<135 kg of BW; n = 108); ^2^ SEM: standard error of the mean; ^3^ Total born refers to the average of total born per farrowing over their lifetime; ^4^ Percentage of sows producing 40 piglets weaned over three parities; ^5^ Data analyzed by logistic regression. a, b Means within a row with different letters were significantly different at *p* ≤ 0.05.

## Data Availability

The datasets generated during and/or analyzed during the current study are available from the corresponding author (S.M.) on reasonable request.
